# Kidney-specific claudin-2 deficiency leads to medullary nephrocalcinosis in mice

**DOI:** 10.1172/JCI197807

**Published:** 2025-10-09

**Authors:** Christine V. Behm, Duuamene Nyimanu, Ony Araujo Galdino, Sadhana Kanoo, Young Chul Kim, Natalia Lopez, Helen Goodluck, Peter S. Rowe, Andrew P. Evan, André J. Sommer, Matthew N. Barr, Tracy Punshon, Volker Vallon, Brian P. Jackson, James C. Williams, Alan S.L. Yu

**Affiliations:** 1 Division of Nephrology and Hypertension, Department of Internal Medicine, and Jared Grantham Kidney Institute, University of Kansas Medical Center, Kansas City, Kansas, USA.; 2 Division of Nephrology and Hypertension, Department of Medicine, University of California San Diego, La Jolla, California.; 3 VA San Diego Healthcare System, San Diego, California, USA.; 4 Department of Anatomy, Cell Biology and Physiology, Indiana University School of Medicine, Indianapolis, Indiana, USA.; 5 Biomedical National Elemental Imaging Resource, Department of Earth Sciences, Dartmouth College, Hanover, New Hampshire, USA.

**Keywords:** Metabolism, Nephrology, Calcium, Epithelial transport of ions and water, Tight junctions

## Abstract

Deposits of hydroxyapatite called Randall’s plaques are found in the renal papilla of calcium oxalate kidney stone formers and likely serve as the nidus for stone formation, but their pathogenesis is unknown. Claudin-2 is a paracellular ion channel that mediates calcium reabsorption in the renal proximal tubule. To investigate the role of renal claudin-2, we generated kidney tubule–specific claudin-2 conditional KO mice (KS-Cldn2 KO). KS-Cldn2 KO mice exhibited transient hypercalciuria in early life. Normalization of urine calcium was accompanied by a compensatory increase in expression and function of renal tubule calcium transporters, including in the thick ascending limb. Despite normocalciuria, KS-Cldn2 KO mice developed papillary hydroxyapatite deposits, beginning at 6 months of age, that resembled Randall’s plaques and tubule plugs. Bulk chemical tissue analysis and laser ablation–inductively coupled plasma mass spectrometry revealed a gradient of intrarenal calcium concentration along the corticomedullary axis in normal mice that was accentuated in KS-Cldn2 KO mice. Our findings provide evidence for the “vas washdown” hypothesis for Randall’s plaque formation and identify the corticomedullary calcium gradient as a potential target for therapies to prevent kidney stone disease.

## Abstract 2

Knockout of the claudin-2 gene in kidneys of mice leads to increase in medullary calcium concentration gradient and deposition of mineral in the renal papilla.

## Graphical abstract

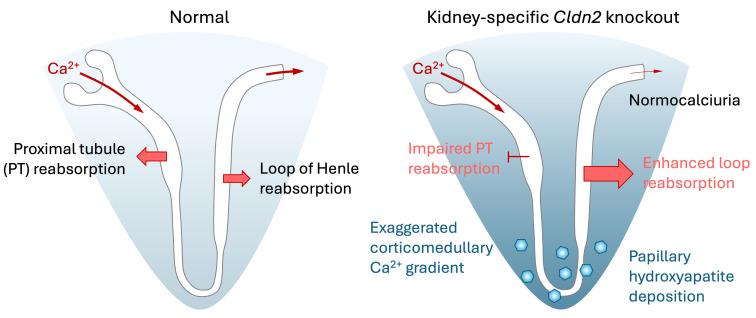

## Introduction

The incidence of kidney stone disease has been increasing steadily, and lifetime risk is now estimated to exceed 22% in males and 15% in females (1). The most common type of kidney stone is calcium oxalate, and idiopathic hypercalciuria (IH) is its major risk factor (2). IH is heritable and polygenic and results from the combined effect of intestinal hyperabsorption of calcium (3), renal calcium wasting (4, 5), and bone calcium resorption (6). An important initial pathogenic step is the deposition in the medullary interstitium of calcium phosphate mineral in the form of hydroxyapatite, known as Randall’s plaques (7–9). These grow over time and rupture through the papillary epithelium into the urinary space, exposing a nidus for the precipitation and growth of calcium oxalate stones.

In the kidney, the majority of filtered calcium (70%) is reabsorbed in the proximal tubule (PT). Transcellular reabsorption of sodium and water in the PT leads to a rise in the luminal calcium concentration that, together with the lumen-positive transepithelial voltage in the late PT, provides the driving force for passive diffusion of calcium via the paracellular pathway (10, 11). Claudins are tight junction membrane proteins that function as paracellular channels. Claudins 2 and 12 are the predominant calcium-permeable paracellular channels in the PT. Global, constitutive KO of the claudin-2 gene (*Cldn2*) in mice (12), or double KO of both *Cldn2* and *Cldn12* (13), leads to hypercalciuria.

Curry et al. showed that the hypercalciuria in constitutive *Cldn2*-KO mice was caused by both renal calcium leak and primary intestinal hyperabsorption of calcium (12). Constitutive *Cldn2*-KO mice also exhibited papillary deposits of hydroxyapatite. Thus, constitutive *Cldn2*-KO mice phenocopy the early pathogenic steps in human IH and kidney stone disease. Furthermore, common, noncoding variants in the human *CLDN2* gene were found to be associated with an increased risk of kidney stone disease in the general population, and a rare missense mutation in *CLDN2* was discovered in one family with an X-linked syndrome of hypercalciuria, kidney stone disease, and male infertility (12).

Claudin-2 is highly expressed not only in the kidney, but also throughout the intestinal epithelium. To elucidate the role of kidney claudin-2 in the pathogenesis of hypercalciuria and kidney stone disease, we generated floxed *Cldn2* mice and developed renal tubule-specific *Cldn2* conditional KO mouse models.

## Results

### Generation and characterization of kidney-specific claudin-2–KO mice.

**Figure 1:**
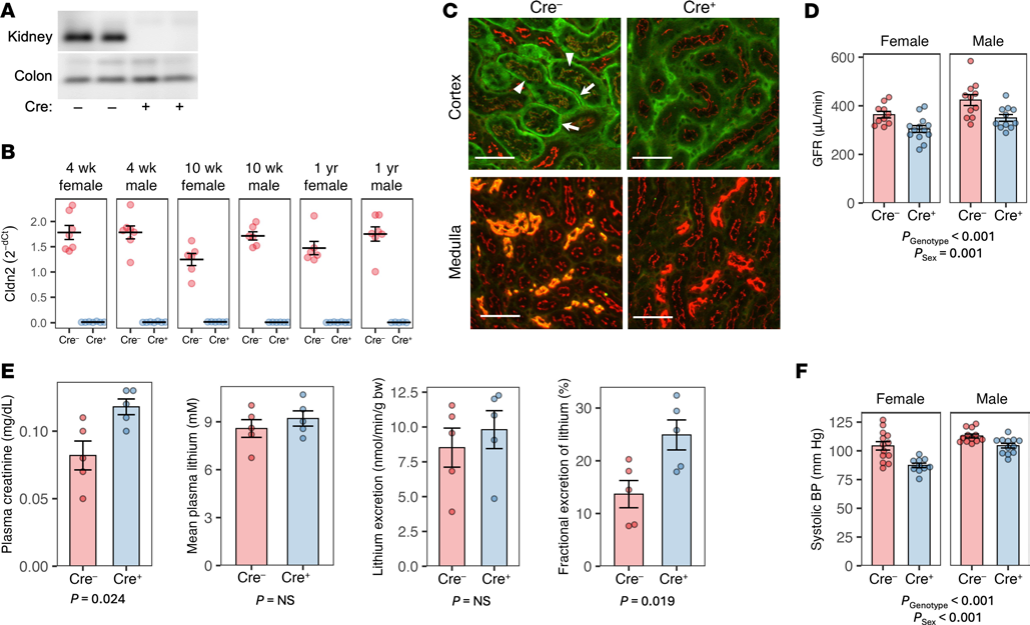
General phenotype of constitutive, kidney-specific *Cldn2*-KO mice. (**A**) Western blot for claudin-2 in kidney and colon tissue from KO mice (Cre^+^) and control littermates (Cre^–^). (**B**) *Cldn2* mRNA expression by quantitative PCR in kidneys, relative to ezrin, plotted as 2^–ΔCt^ values. Differences are significant for genotype (*P* < 0.001), age (*P* = 0.03 for 10 vs. 4 weeks), and sex (*P* = 0.04) by 3-way ANOVA. (**C**) Immunofluorescence staining of kidney cortex and medulla with antibodies to claudin-2 (green) and ZO-1 (red). Orange/yellow fluorescence indicates colocalization of claudin-2 with ZO-1. Claudin-2 is detectable at the tight junctions (arrowheads) and basolateral membrane (arrows) of PTs in the cortex, and in thin descending limbs in the medulla of Cre^–^ control but not Cre^+^ KO mice. Scale bars: 50 μm. (**D**) GFR determined from FITC-sinistrin clearance. *n* = 10–13 per group. (**E**) Determination of fractional excretion of lithium. Following pretreatment with bumetanide to block thick ascending limb sodium and lithium transport, male mice were given an i.v. bolus of LiCl, and plasma creatinine concentration, plasma lithium (mean of samples 3 minutes and 60 minutes after injection), and lithium excretion in a 1-hour urine collection were determined. *n* = 5 per group. (**F**) Systolic BP determined by tail cuff measurement. *n* = 10–13 per group.

We crossed *Cldn2* floxed mice with Pax8-Cre mice to generate constitutive kidney-specific *Cldn2*-KO mice. *Cldn2* was efficiently deleted from both the PT and thin descending limbs (Figure 1, A–C). Claudin-2 has been shown to mediate PT paracellular Na^+^ reabsorption (14). Consistent with this, glomerular filtration rate (GFR) was reduced in the KO mice compared with control littermates (Figure 1D), presumably due to increased Na^+^ delivery to the macula densa, activating tubuloglomerular feedback. We used clearance of lithium, a monovalent alkali cation like Na^+^ that is reabsorbed predominantly in the PT, to assess PT reabsorptive function. Urinary excretion of lithium was not different between KO mice and controls, but because GFR was reduced, fractional excretion of lithium was increased (Figure 1E). This demonstrated that PT reabsorption of Na^+^, and potentially other cations, was impaired with *Cldn2* KO. Systolic blood pressure was also reduced in the *Cldn2*-KO mice (Figure 1F), presumably due to loss of total body Na^+^.

### Kidney-specific claudin-2 KO causes transient hypercalciuria.

**Figure 2:**
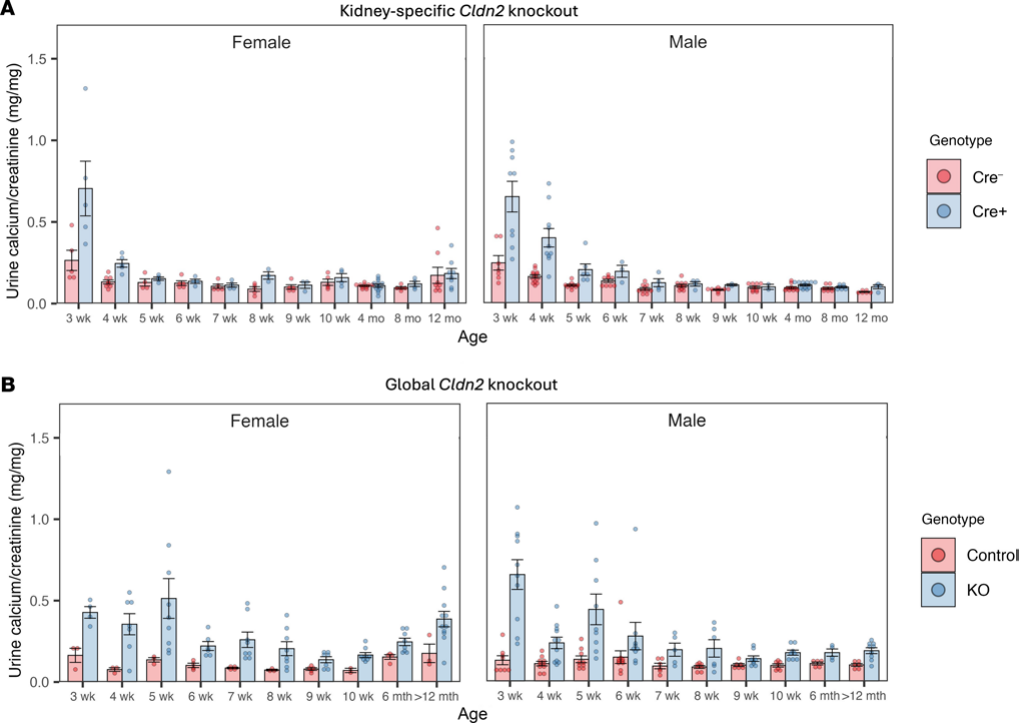
Elevated urine calcium excretion in young but not older *Cldn2*-KO mice. (**A**) Urine calcium/creatinine ratio in kidney-specific *Cldn2*-KO mice. Levels are higher in Cre^+^ compared with Cre^–^ mice (least squares mean difference ± SE = 0.143 ± 0.046, *P* = 0.002) and decrease more rapidly with age (*P* = 0.002 for genotype × age interaction), with no difference between the sexes, using LMM. (**B**) Urine calcium/creatinine ratio in global, constitutive *Cldn2*-KO mice. Levels are higher in KO mice than control littermates (least squares mean difference ± SE = 0.193 ± 0.039, *P* < 0.001), but there is no significant interaction with age. Age group >12 months included mice ranging from 63 to 101 weeks old. *n* = 2–13 per group.

To test the hypothesis that claudin-2 in the renal tubule regulates urine calcium excretion, we measured urine calcium excretion in constitutive kidney-specific *Cldn2*-KO mice. Constitutive KO mice were hypercalciuric at weaning, but their urine calcium level decreased with age and was similar to that in controls by 6–8 weeks (Figure 2A). By contrast, global, constitutive *Cldn2*-KO mice, which we previously reported to be hypercalciuric (12), showed increased urine calcium throughout adulthood up to 2 years of age (Figure 2B). This suggests that kidney-specific *Cldn2*-KO mice are able to fully compensate for the defect in paracellular calcium reabsorption with age, whereas global *Cldn2*-KO mice are not.

**Figure 3:**
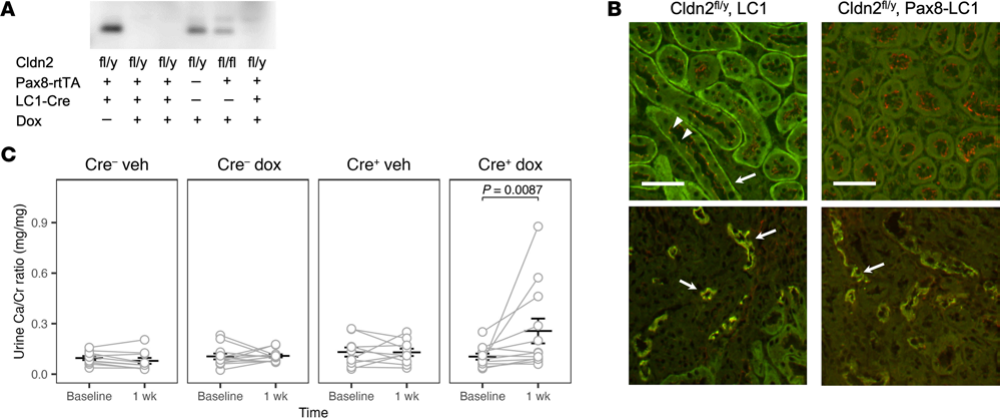
Induction of hypercalciuria within a week in inducible kidney-specific *Cldn2*-KO mice. (**A**) Western blot for claudin-2 in whole kidney lysates from KO mice (*Cldn2^fl/y^*, Pax8-LC1) treated with doxycycline (Dox) or vehicle (lane 1) and control littermates without the Pax8 and/or LC1 genes (lanes 4 and 5). (**B**) Immunofluorescence staining of kidney sections with antibodies to claudin-2 (green) and ZO-1 (red). The upper panels show kidney cortex, where claudin-2 is detectable at the tight junctions (arrowheads) and basolateral membrane (arrow) of PTs in the control mouse (left) and absent in the KO mouse (right). The lower panels show the inner stripe of outer medulla, where claudin-2 localized to the thin descending limbs (arrows) is only mildly reduced in the KO compared with control. Claudin-2 deletion in the thin descending limbs was incomplete even after 3 weeks of doxycycline induction (not shown). Scale bars: 50 μm. (**C**) Urine calcium/creatinine (Ca/Cr) ratio before and after 1 week of doxycycline or vehicle (Veh) in inducible KO mice (Cre^+^) or control littermates without LC1 (Cre^–^). Bars represent mean ± SEM. *P* value is shown for interaction of time with the group Cre^+^ Dox, by repeated-measures LMM.

To confirm that deletion of *Cldn2* causes hypercalciuria due to a functional transport defect and not due to a developmental abnormality, we generated inducible kidney-specific *Cldn2*-KO mice by crossing claudin-2 floxed mice to Pax8-LC1 (“Tet-On”) mice (Supplemental Figure 1; supplemental material available online with this article; https://doi.org/10.1172/JCI197807DS1). After induction with oral doxycycline for 1 week, claudin-2 protein was no longer detectable in whole kidney lysates by immunoblotting or in PT by immunofluorescence, whereas claudin-2 was well expressed in control mice with omission of the Pax8 or LC1 transgene or treated with vehicle (Figure 3, A and B). Claudin-2 is normally also expressed in the upper segments of the thin descending limbs of long-looped nephrons. However, in these mice doxycycline induced only a partial deletion of *Cldn2* from thin descending limbs (Figure 3B), which was due to inefficient Cre recombination (Supplemental Figure 2). Nevertheless, treatment of 5-week-old inducible KO mice with 1 week of doxycycline induced a 2.5-fold increase in urine calcium/creatinine ratio, demonstrating that PT claudin-2 was essential for tubular calcium reabsorption (Figure 3C).

### Compensatory upregulation of parathyroid hormone and thick ascending limb and distal convoluted tubule calcium transport mechanisms in response to loss of kidney claudin-2.

**Figure 4:**
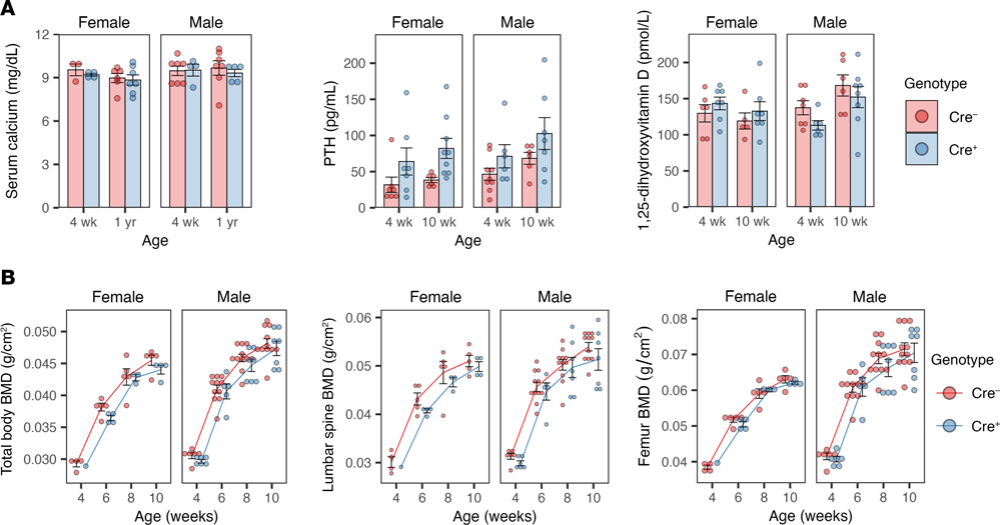
Analysis of response to hypercalciuria in constitutive kidney-specific *Cldn2*-KO mice shows compensatory upregulation of PTH. (**A**) Serum calcium, intact PTH, and 1,25-dihydroxyvitamin D levels. *n* = 3–9 per group. (**B**) Total body, lumbar spine, and femur bone mineral density (BMD) between 4 and 10 weeks of age. *n* = 4–11 in all groups except 4-week-old Cre^+^ female (*n* = 1). *P* = 0.0008 for the effect of genotype on PTH levels by 3-way ANOVA. For all other measures, *P* is nonsignificant for genotype and its interactions with sex and age.

We previously showed (12) in global claudin-2–KO mice that renal calcium loss was matched by primary intestinal hyperabsorption of calcium, leading to even total body calcium balance. We therefore predicted that KO of *Cldn2* in the renal tubule but not the intestine would lead to negative calcium balance and be reflected in compensatory hormonal changes. We found that serum calcium was unchanged (Figure 4A), but parathyroid hormone (PTH) was increased in kidney-specific *Cldn2*-KO mice by 1.5-fold (males) to 2-fold (females) compared with controls (Figure 4B, *P* = 0.0008), as would be expected in response to calcium wasting. There was no change in 1,25-dihydroxyvitamin D levels (Figure 4C). Bone mineral density at the lumbar spine and femur, or summed over the whole body (Figure 4D), tended on average to be lower in the KO mice at all ages, but the differences did not reach statistical significance.

**Figure 5:**
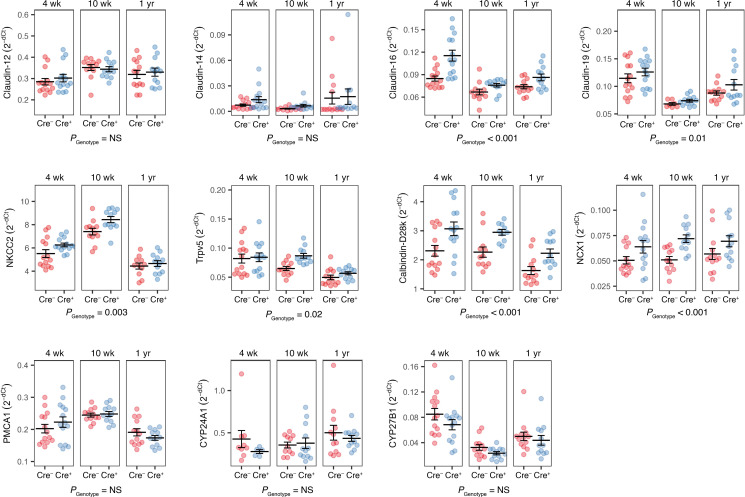
Compensatory upregulation of mRNA expression of thick ascending limb and distal convoluted tubule calcium transporters in constitutive kidney-specific *Cldn2*-KO mice. Expression levels for each gene by quantitative PCR, relative to ezrin, are plotted as 2^–ΔCt^ values. *P* values are reported for differences between genotype (Cre^+^ vs. Cre^–^) by 3-way ANOVA with between-group factors of age, sex and genotype. Sex as a factor was not significant, so males and females are grouped for presentation. *n* = 6–7 per age group and genotype.

To determine whether defective PT calcium reabsorption was compensated by upregulation of other calcium transporters, we quantitated their mRNA levels by quantitative PCR (qPCR) at 4 weeks (when the kidney-specific *Cldn2*-KO mice were hypercalciuric), 10 weeks (when they were no longer hypercalciuric), and 1 year of age (Figure 5). The major PTH-regulated calcium transport proteins in the distal convoluted tubule — TrpV5, calbindin D28k, and NCX1 (15) — were all significantly upregulated in kidney-specific *Cldn2*-KO mice. Likewise, the major calcium transport proteins in the thick ascending limb of Henle (NKCC2, claudin-16, and claudin-19) were significantly upregulated. Although claudin-12 has been shown to contribute to renal calcium reabsorption in the PT (13), we observed no compensatory upregulation of claudin-12. There was also no change in the expression of claudin-14, the plasma membrane calcium ATPase PMCA1, or the hydroxylase enzymes for activation and inactivation of vitamin D (Cyp27b1, Cyp24a1).

**Figure 6:**
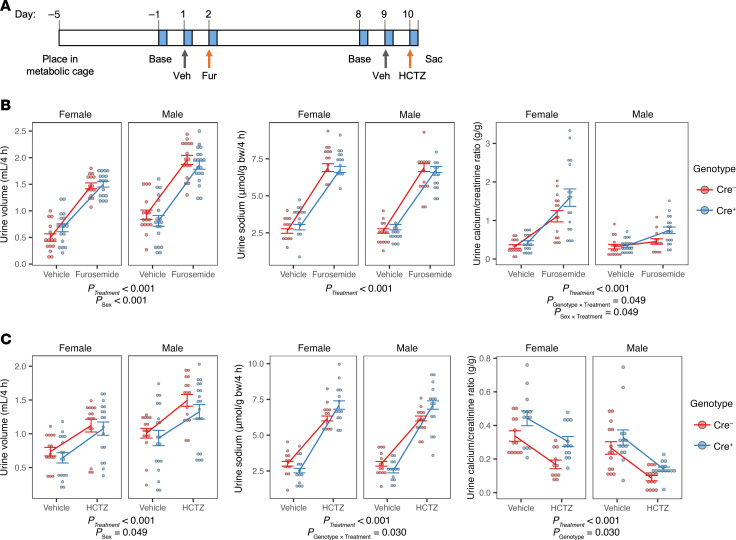
Diuretic challenge assay unmasks enhanced thick ascending limb calcium reabsorption in constitutive kidney-specific *Cldn2*-KO mice. (**A**) Diagram of experimental protocol. Four-hour urine collections (blue) were taken at baseline (Base) a day prior to sequential injections with vehicle, followed the next day with diuretic, either furosemide (Fur) or hydrochlorothiazide (HCTZ). The 2 diuretics were separated by an interval of 1 week. Diet was switched to gel formulation on day –1 to ensure adequate hydration. Effect of furosemide (**B**) and hydrochlorothiazide (**C**) compared with vehicle on urine volume, sodium excretion, and calcium excretion. Urine measurements were modeled by LMM with between-subject effects of genotype and sex, within-subject effect of treatment, and the interaction of genotype and sex with treatment. *n* = 15–19 per group. The statistically significant fixed effects are listed below each panel.

To test functionally for compensatory upregulation of reabsorption in the thick ascending limb of Henle and the distal convoluted tubule, we performed diuretic challenge assays in 12 week-old mice using a loop diuretic, furosemide; and a thiazide diuretic, hydrochlorothiazide (Figure 6A). Over the 4-hour period after furosemide administration, urine volume and urine sodium excretion were increased equally in kidney-specific *Cldn2*-KO and control mice (Figure 6B). Urine calcium excretion was also increased with furosemide, with female mice exhibiting greater calciuresis. Importantly, calcium excretion was increased to a greater extent in kidney-specific *Cldn2*-KO mice compared with controls, with a least-squares mean difference (±SE) of 0.39 ± 0.19 g/g (*P* = 0.039 for genotype × treatment interaction).

The increase in sodium excretion with hydrochlorothiazide treatment compared with vehicle was greater in the KO mice than controls, with a least-squares mean difference (±SE) of 1.32 ± 0.59 μmol/g body weight/hour (*P* = 0.03 for genotype × treatment interaction). Hydrochlorothiazide is not expected to block Ca reabsorption in distal convoluted tubule and actually decreased urine calcium excretion, with no significant difference between KO and control mice (Figure 6C).

### Nephrocalcinosis in older mice despite normocalciuria.

**Figure 7:**
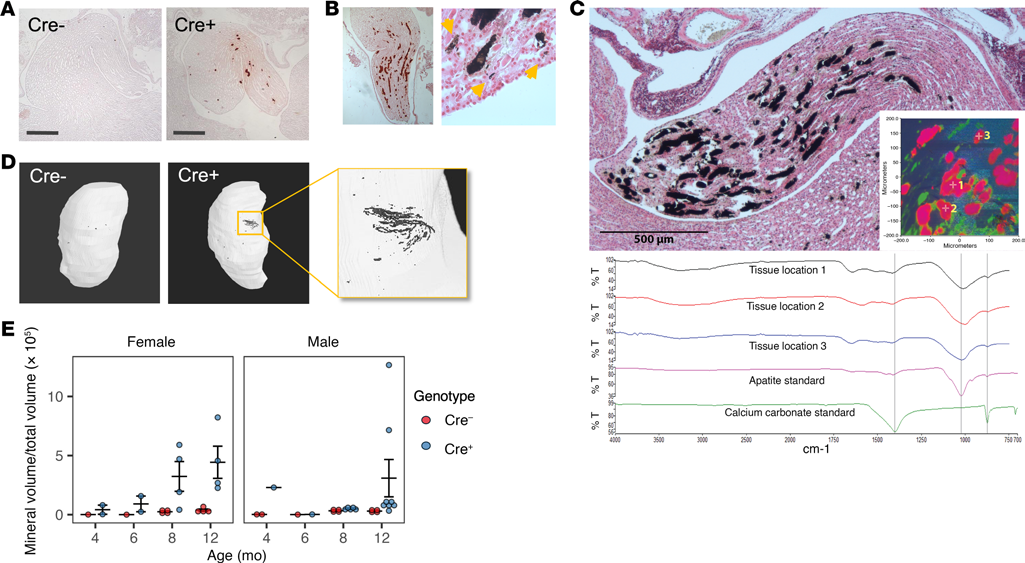
Nephrocalcinosis in older kidney-specific *Cldn2*-KO mice. (**A**) Alizarin red staining of the renal inner medulla of 1-year-old female Cre^–^ and Cre^+^ mice. Scale bar: 400 µm. (**B**) Left: Papilla of a 16 month-old female Cre^+^ mouse stained with alizarin red. Right: High-magnification view of Yasue-stained section showing large intratubular plugs and smaller interstitial granular deposits (yellow arrows). (**C**) Infrared analysis of mineral deposits. Top panels: Yasue-stained section showing extensive mineral deposition in renal papilla. Inset: False-color representation of spectrum field with infrared microscope; square is 400 x 400 μm. Bottom panels: Spectra from mineral locations 1–3, indicated in the inset, along with standard spectra for apatite and calcite (calcium carbonate). (**D**) Micro-CT scans of the kidneys from **A** at bone density setting to detect mineral deposits. (**E**) Quantitation of mineral volume as a proportion of total kidney volume (*P* = 0.0036 for effect of genotype by 3-way ANOVA).

We previously reported that global *Cldn2*-KO mice, which are hypercalciuric throughout life, exhibit papillary nephrocalcinosis (12). Surprisingly, despite the fact that kidney-specific *Cldn2-*KO mice were normocalciuric throughout their adult life, they also developed medullary nephrocalcinosis. By histological staining and micro-computed tomography scanning, we observed linear inner medullary deposits of calcium mineral, densely concentrated in the papilla, that began to appear from 6 to 8 months of age in females and 12 months in males (Figure 7). A majority of deposits were intraluminal, while a few large, flat-shaped deposits were seen in the interstitium (Figure 7C). By infrared spectroscopy, the composition of the deposits was determined to be predominantly calcium phosphate in the form of apatite, with some calcium carbonate (Figure 7D). Importantly, this occurred despite KS-*Cldn2* KO mice being normocalciuric from 6 weeks of age. Thus, there was a striking dissociation in these mice between urine calcium excretion and kidney tissue mineral deposition.

### Discovery of a corticomedullary calcium gradient.

The finding that kidney-specific *Cldn2-*KO mice developed papillary nephrocalcinosis despite normocalciuria suggests that their normal urine calcium might be masking an accumulation of calcium within the medullary tissue. Our finding that these mice have compensatory thick ascending limb reabsorption of calcium provides the mechanistic basis for this. Mathematical modeling has predicted the existence of a corticomedullary interstitial gradient of calcium in the normal kidney (16). Moreover, in IH stone-formers, who also have a defect in PT calcium reabsorption (17–19), it has been hypothesized that the increased delivery of calcium from the PT to the loop of Henle — and the increased reabsorption of calcium from the thick ascending limb — leads to greater concentration of calcium in the inner medullary interstitium and papilla due to the vasa recta blood flow and countercurrent exchange, which has been called the “vas washdown” hypothesis (20).

**Figure 8:**
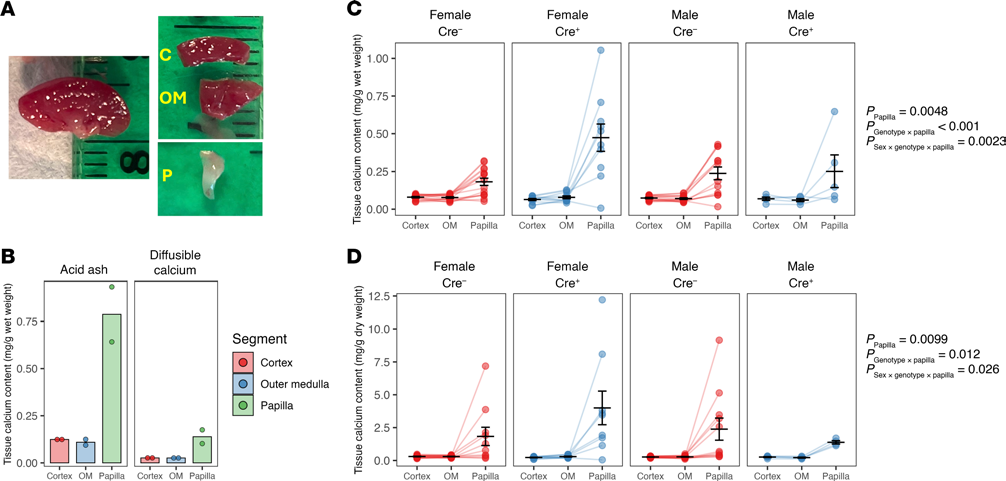
Enhanced papillary Ca content in 4- to 6-month-old kidney-specific claudin-2–KO mice. Bulk chemical analysis of the axial distribution of tissue calcium in mouse kidney. (**A**) Dissection of mouse kidney. Coronal section through kidney on left; and dissection of 3 regions on right. C, cortex; OM, outer medulla and base of inner medulla; P, papilla. Small tick marks on the ruler represent 1 mm intervals. (**B**) Comparison of 2 methods of tissue calcium extraction (acid ash and diffusible calcium) in WT mice. (**C** and **D**) Kidney calcium content relative to tissue wet weight (**C**) or dry weight (**D**) in kidney-specific claudin-2–KO mice (Cre^+^) as compared with control littermates (Cre^–^). Data were analyzed with multilevel LMMs, with kidney region as the level 1 unit, nested within individual mice as level 2. Statistically significant *P* values are reported for the fixed effects of kidney region (papilla or outer medulla, relative to cortex as the reference), genotype, and sex and their interactions. *n* = 3–9 mice per group.

To test this hypothesis, we determined the axial distribution of calcium content in the kidney in mice at a sufficiently early age that mineral deposits were not yet detectable (16–17 weeks). The kidneys were dissected into cortex, medulla, and papilla, bulk tissue calcium was extracted by 2 different methods, and calcium content was quantitated (Figure 8A). Calcium concentration using the acid ash method, which measures total mineral content, was approximately 5-fold higher than by the diffusible calcium method. However, the 2 methods revealed a similar relative calcium distribution, with higher calcium concentration in the papilla than in the cortex and medulla (Figure 8B). Given the decreased PT calcium reabsorption in kidney-specific *Cldn2*-KO mice and compensatory upregulation of the function and expression of the thick ascending limb calcium transporters, we predicted that the corticomedullary calcium gradient would be exacerbated. Indeed, we found that papillary calcium concentration was greater in kidney-specific *Cldn2*-KO mice than in controls. This was more marked in female mice (least-squares mean difference (±SE) of 0.290 ± 0.043 mg/g wet weight, *P* < 0.0001) and significantly attenuated in males (least-squares mean difference ± SE of 0.014 ± 0.056 mg/g wet weight, *P* = 0.8). Moreover, the results were similar whether calcium content was expressed relative to wet weight or to dry weight of tissue (Figure 8, C and D). In older animals (26 weeks), the accumulation of papillary calcium in kidney-specific *Cldn2*-KO mice was even more striking and occurred independent of sex (Supplemental Figure 3).

### Spatial distribution of intrarenal calcium.

To visualize the 2D spatial distribution of calcium within the kidney at higher resolution, we performed elemental analysis on flash-frozen mouse kidney sections by laser ablation–inductively coupled plasma time-of-flight mass spectrometry (LA-ICP-TOFMS). This is a highly sensitive analytical technique in which a laser scans small areas of the tissue section, creating an aerosol that is then introduced into high-temperature plasma. The plasma vaporizes and ionizes the particles, and the mass spectrometer separates and detects these ions based on their mass-to-charge ratio.

**Figure 9:**
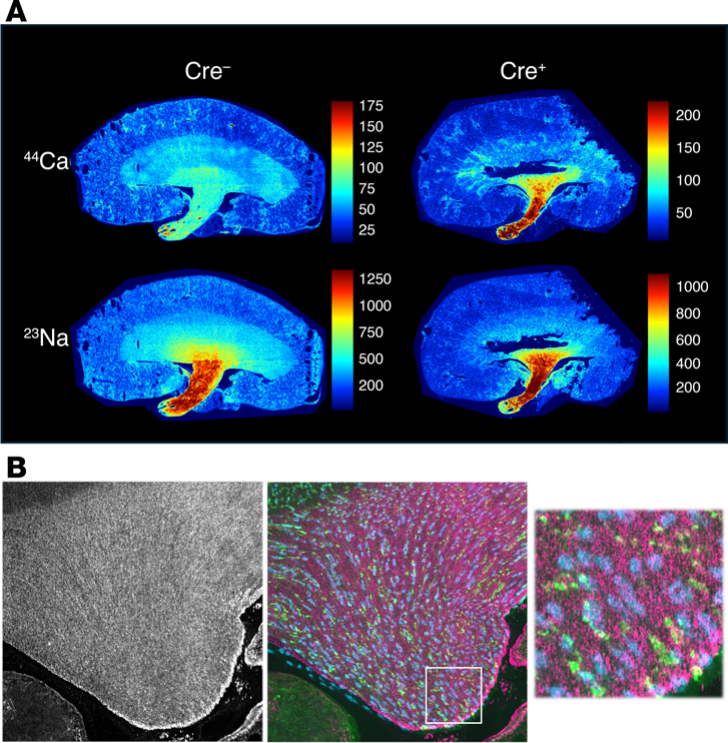
Elemental mapping by LA-ICP MS in 6-week-old mouse kidneys. (**A**) 2D maps of calcium (^44^Ca) and sodium (^23^Na) in midline coronal kidney sections of *Cldn2* kidney-specific KO (Cre^+^) and control (Cre^–^) mice. Color scale represents concentration in ppm. (**B**) High-resolution image of a Cre^+^ mouse papilla. Left*:* ^44^Ca map at 2 μm resolution. Middle*:* Overlay of ^44^Ca map (magenta) with immunofluorescence labeling of an adjacent section with AQP1 (green) and AQP2 (cyan). Region delineated by the white square is magnified in the right panel. The appearance of a stippled pattern in the calcium channel is consistent in size with individual laser spots and indicates that calcium is homogeneously distributed.

**Figure 10:**
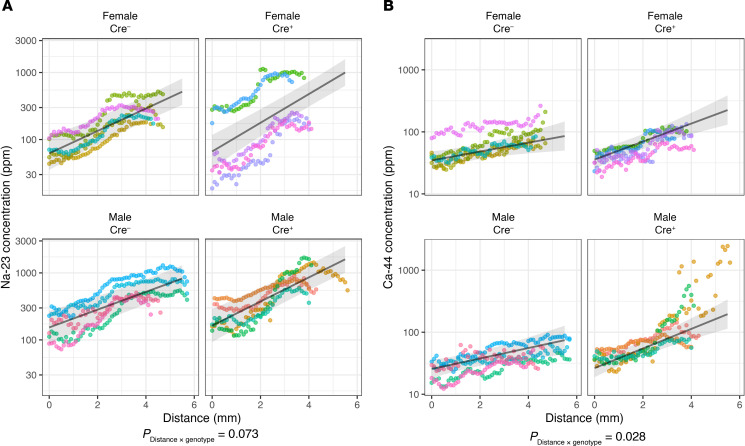
Elemental corticomedullary concentration profiles. Profiles for ^23^Na (**A**) and ^44^Ca (**B**). Element concentrations in parts per million (ppm) are plotted on a log scale. Distance is measured from the cortical surface in millimeters. Kidney sections from each individual mouse are represented by a different color (*n* = 4–5 mice per group). Black lines and shaded bands represent the LMM predictions from marginal means and their 95% confidence intervals. *P* values are reported for slope of log-concentration versus distance.

Studies were performed in mice at 6 weeks of age, before the development of mineral deposits. The distribution of sodium, which is known to exhibit a corticomedullary gradient, was used as a positive control. A corticomedullary gradient for calcium was observed, and the gradient for sodium was confirmed (Figure 9A and Supplemental Figures 4 and 5). By contrast, phosphorus concentration was uniformly distributed throughout the kidney (Supplemental Figures 6 and 7) and other elements showed distinctly different distributions (Supplemental Figure 7). The ratio of the calcium concentration at the papillary tip to that in the superficial cortex was 3.36 ± 0.50 (mean ± SD) in control (Cre^–^) mice and 15.20 ± 32.23 (mean ± SD) in kidney-specific *Cldn2*-KO (Cre^+^) mice (Supplemental Table 1). Calcium appeared to increase exponentially with distance from cortex to medulla (Figure 10). When log-transformed calcium concentration was fitted to a linear mixed model, the calcium concentration slope as a function of distance was approximately 2-fold greater in Cre^+^ compared with Cre^–^ mice (*P* = 0.02 for the distance × genotype interaction; Supplemental Table 2). With high-resolution mapping (Figure 9B), the calcium distribution in the papilla did not localize to tubule- or vascular-like structures, but appeared homogeneous. Overlay with immunofluorescently stained adjacent sections for the collecting duct (aquaporin 2 [AQP2]) and descending thin limbs (aquaporin 1 [AQP1]) did not show any suggestion of selective colocalization.

## Discussion

In summary, we have created a mouse model with conditional kidney-specific KO of claudin-2 that exhibits medullary deposition of hydroxyapatite despite normocalciuria. Our findings suggest that impaired PT paracellular calcium reabsorption in these mice is compensated by increased PTH secretion and by upregulation of transcellular and paracellular calcium transporters in the thick ascending limb, as well as the distal convoluted tubule. Our data are consistent with the hypothesis that increased tubule delivery of calcium to the loop of Henle and compensatory increase in reabsorption of calcium into the medullary interstitium is maladaptive and leads to development of medullary nephrocalcinosis.

This mouse model illustrates a dissociation between urine calcium excretion and kidney tissue mineral deposition. Hypercalciuria and increased urinary supersaturation of calcium salts is conventionally thought to be the major causal risk factor for nephrocalcinosis. However, in contrast to claudin-2–KO mice, mice with genetically defective calcium reabsorption in the thick ascending limb and distal convoluted tubule have little or no nephrocalcinosis despite much more severe hypercalciuria (21–25) (Supplemental Table 3). Likewise, rats bred for 56 generations to excrete 8–10 times as much urine calcium as controls nevertheless exhibit no kidney mineral deposits whatsoever (26). Conversely, thick ascending limb–specific KO of claudin-10 reduces fractional excretion of calcium, presumably by increasing thick limb reabsorption of calcium, and leads to outer medullary nephrocalcinosis despite hypocalciuria (27). These observations are all consistent with a model in which reabsorption of calcium in the loop of Henle is the key determinant of nephrocalcinosis. We suggest that the PT site for the defect in calcium transport in claudin-2 mice is unique in increasing delivery of calcium to the loop of Henle, where it both accumulates in the lumen and is reabsorbed and deposited in the interstitium.

A key prediction of our pathogenic model was that the kidneys would have a corticomedullary interstitial gradient for calcium, analogous to the gradient for sodium chloride and urea. The existence of such a gradient in the normal kidney was previously predicted by mathematical modeling (16). Moreover, reports of regional calcium content measurements in the older literature in dogs (28) and in human kidneys (29, 30) suggested that the normal renal papilla is enriched in calcium. In our study, we were able to confirm this and visualize a corticomedullary gradient for calcium using 2 complementary approaches. Bulk tissue chemical analysis showed higher calcium concentration in the papilla, while spatial fine mapping by mass spectrometry showed a continuous axial corticomedullary calcium gradient. Our method of bulk chemical analysis was clearly very crude compared with LA-ICP-TOFMS, which may well explain the different results. The preparation time (and hence time for diffusion) was longer (2 minutes of dissection vs. snap freezing tissue in seconds), the spatial resolution was poorer (millimeter vs. 2–6 μm resolution), and the sensitivity was lower (50 ppm vs. sub-ppm). Additionally, we hypothesized that claudin-2–KO mice would have increased papillary calcium sequestration, which was indeed the case.

Our findings are important because we believe claudin-2–KO mice model the development of Randall’s plaques, which is a key step in the pathogenesis of human calcium oxalate urinary stones (31, 32). The mineral deposits in claudin-2–KO mice, like Randall’s plaques, are concentrated in the papilla and composed predominantly of hydroxyapatite (8, 33). Like claudin-2–KO mice, kidney stone formers with idiopathic hypercalciuria have a defect in PT calcium reabsorption (17, 19). The “vas washdown” model hypothesizes that increased calcium delivery to the loop leads to increased reabsorption of calcium into the medullary interstitium, where the descending vasa recta serve to convey that calcium toward the inner medulla, raising the supersaturation of calcium phosphate to a sufficiently high level in the papilla to deposit as hydroxyapatite (34). The credibility of this model is bolstered by the observation that multiple common variants and 1 rare missense mutation in the human claudin-2 gene are associated with kidney stone disease (12). The implication of this is that the pathways that lead to papillary calcium deposition may be therapeutic targets for kidney stone disease.

One caveat to this is that our mice differ from the canonical description of Randall’s plaques in idiopathic hypercalciuric stone formers in that they have not only medullary interstitial hydroxyapatite deposits but also tubule plugs. Moreover, when we examine mice by LA-ICP-MS before the development of hydroxyapatite deposits, the distribution of papillary calcium appeared homogeneous, suggesting that the increased calcium concentration in the papilla is equilibrated between the interstitium and the tubules. This is not altogether surprising, since our model predicts that there should be more calcium delivered to *both* the tubule lumen of the loop of Henle and the surrounding medullary interstitium. Moreover, even in calcium oxalate stone formers, interstitial plaques have been reported to frequently co-occur with intratubular plugs of mineral (35, 36).

A number of interesting observations in our study deserve brief comment. We found that doxycycline induction of the Pax8-LC1 Cre (sometimes referred to as Pax8rtTA;TetO-Cre) was incomplete and excluded the thin descending limb. Despite this, inducible claudin-2 KO had robust hypercalciuria. This is consistent with the accepted view that the thin descending limb is impermeable to calcium and presumably plays minimal role in calcium reabsorption (37). However, it raises the intriguing question of what the functional role of claudin-2 is in this nephron segment, given that it is the site with the highest level of claudin-2 expression (38).

Our finding that the kidney-specific claudin-2–KO mice were able to compensate for defective PT calcium reabsorption and normalize urine calcium by 6–8 weeks of age, whereas the global KO mice could not, supports the idea that the global KO mice had intestinal hyperabsorption of calcium and were in positive calcium balance so that PTH and other compensatory mechanisms were in part suppressed. That the kidney-specific KOs were transiently hypercalciuric early in life may have been due to the sequelae of the large calcium load from suckling, developmental changes in calcium transporter expression, or just the time taken for compensatory increases in gene expression to occur. We did not directly test the mechanism for upregulation of expression of calcium transport proteins in kidney-specific claudin-2–KO mice, but several are known to be upregulated by PTH, including TrpV5, calbindin D28k, and NCX1 (15).

We did not identify any significant sex differences in hypercalciuria in our mice. Claudin-2 expression is higher in males than females (39, 40), and we also observed this in our mice once they reached adulthood (Figure 1B). Thus, one might expect more severe consequences of gene KO in male mice, including worsened hypercalciuria and, if there is more delivery of calcium to the loop, more nephrocalcinosis, but this was not the case. In fact, papillary mineral deposition occurred earlier in female KO mice. One explanation for this is that the burden of sodium reabsorption is redistributed from the proximal to the distal nephron in females (40). We observed a nonsignificant trend toward greater natriuresis in response to furosemide in females compared with males and significantly greater calciuresis (Figure 6B). This suggests that increased reabsorption of calcium in the outer medulla may lead to earlier accumulation of interstitial deposits in the female mice. The clinical significance of this is uncertain, however, as we found no sex difference in the association of *CLDN2* common variants with kidney stones in our previous genome-wide association study (12).

We had predicted that the kidney-specific KO mice would be in negative calcium balance, which would be evident from higher PTH and 1,25-vitamin D levels and lower bone mineral density. We did indeed observe a clear increase in PTH, but 1,25-vitamin D levels were not elevated. This has also been observed in claudin-2 and claudin-12 double-KO mice, despite more severe hypercalciuria, and raises the intriguing question of whether claudin-2 in the PT is needed in some way for 1-hydroxylation of 25-hydroxyvitamin D (13). The lack of significant depletion of bone density is not too surprising. Kidney-specific claudin-2–KO mice are hypercalciuric very transiently in early life and so their cumulative total body calcium deficit is likely to be quite minimal.

The main strengths of our study are that we are able to isolate the role of claudin-2 in the renal tubule by using tissue-specific gene KO, and we could corroborate our findings with 2 different Cre lines. One weakness is that we were unable to perform calcium balance studies because the mice were very young and hence too small to study during the transient period of hypercalciuria.

In conclusion, kidney-specific claudin-2–KO mice were normocalciuric aside from a transient period of hypercalciuria in early life, yet developed papillary nephrocalcinosis with advancing age. We propose that this represents a model of Randall’s plaques and supports the vas washdown theory. Developing therapies to target this mechanism may be an innovative approach to the prevention of kidney stones, and our mice may be a useful model in which to test these.

## Methods

### Sex as a biological variable.

Our study examined male and female animals, and sex-dimorphic effects are reported.

### Generation of kidney-specific claudin-2–KO mouse.

To generate claudin-2 conditional KO mice, we targeted the *Cldn2* gene on the X chromosome by homologous recombination to insert LoxP sites flanking the single coding exon (inGenious), as depicted in Supplemental Figure 1. The mice were then backcrossed for 10 generations onto the C57BL6/J background. Constitutive kidney-specific *Cldn2*-KO mice were generated by crossing *Cldn2* floxed mice (*Cldn2^fl/fl^* or *Cldn2^fl/y^*) with Pax8-Cre knock-in mice, which express Cre constitutively throughout the renal tubule (The Jackson Laboratory) (41). Inducible kidney-specific *Cldn2*-KO mice were generated by crossing *Cldn2* floxed mice with mice carrying transgenes for Pax8-rtTA, which express the reverse tetracycline-dependent transactivator throughout the renal tubule (42), and for TRE-LC1, which express Cre recombinase under the control of an rtTA response element (43), provided by Arohan Subramanya (University of Pittsburgh, Pittsburgh, Pennsylvania, USA) with permission of the German Cancer Research Center (DKFZ). Recombination was induced by feeding doxycycline, 2 mg/mL with 2% sucrose in the drinking water for 7 days. Negative controls were littermates with Pax8 or LC1 omitted, or that were fed 2% sucrose only. Mice were all fed standard lab chow ad libitum (Teklad Rodent Diet 8604, Envigo).

### Immunoblotting, immunofluorescence, and histology.

For protein immunoblots, whole kidney lysates were electrophoresed on sodium dodecyl sulfate-polyacrylamide gels, transferred to polyvinylidene difluoride membrane, and blotted with mouse anti–claudin-2 (Thermo Fisher Scientific 32-5600, 1:500). Immunofluorescence was performed on frozen sections of paraformaldehyde perfusion-fixed kidneys as described previously (12). The primary antibodies used were mouse monoclonal claudin-2 (Thermo Fisher Scientific 12H12) at 1:1,000 for immunoblots, 1:500 for immunofluorescence, and ZO-1 (Santa Cruz Biotechnology SC-337250), 1:500. Histological staining was performed on paraffin-embedded sections with 2% alizarin red S (2% at pH 4.3 for 1–3 minutes) and Yasue metal substitution stain (44).

### Blood and urine assays.

Spot urine specimens were collected by spontaneous voiding onto a Parafilm mat. Mice were then anesthetized with isoflurane and blood was collected by cardiac puncture. Serum and urine calcium were measured by a colorimetric assay (Quantichrom, BioAssay Systems). Urine creatinine concentration was measured by the Jaffe reaction (Cayman Chemical). Plasma intact PTH (MicroVue Mouse PTH 1-84, Quide) and 1,25 dihydroxyvitamin D (Immunodiagnostic Systems) were measured by ELISA.

### Systolic blood pressure and GFR.

These were determined at 12–16 weeks of age. Systolic blood pressure and heart rate were determined in awake mice by an automated tail cuff system (BP-2000 Blood Pressure Analysis System, Visitech Systems) for 6 consecutive days after appropriate training (45, 46). Precautions were taken to reduce the stress of the animals during automated tail cuff blood pressure measurements (47, 48). These included appropriate training of the mice over multiple days, prewarming to an ambient temperature of 29°C, measurement in a quiet, semi-darkened and clean environment, and performance of the measurements by one person and during a defined time of day when blood pressure is stable (between 1:00 and 3:00 pm).

GFR was determined between 9 am and 12 noon using plasma elimination kinetics of FITC-sinistrin (Fresenius-Kabi) measured by a transdermal detection system (NIC-Kidney Device, Medibeacon) (49). Briefly, under short and mild isoflurane anesthesia, a bolus dose of FITC-sinistrin (4%, 2 mL/g body weight in 0.85% NaCl) was injected into the retro-orbital plexus, followed by quick recovery of the mice from anesthesia. The transdermal signal was monitored before FITC-sinistrin injection and over 1.5 hours after injection. Subsequently and after device readout (by MBLAB software, MPD Lab Version 2.2), the data were analyzed and GFR was calculated using manufacturer software (MB Studio2).

### Fractional lithium excretion.

Twenty-week-old male mice were administered the loop diuretic bumetanide to inhibit lithium reabsorption in the thick ascending limb (40 mg/kg body weight i.p.; B3023, Sigma-Aldrich; dissolved in ethanol and diluted in saline 1:10; 10 mL/g body weight). Thirty minutes later, the bladder was emptied by stimulating spontaneous urination and LiCl (5 mmol/kg body weight; 62476, Sigma-Aldrich; 2 mL/g body weight) injected by retro-orbital plexus injection under brief isoflurane anesthesia. The mice were subsequently housed in metabolic cages for timed urine collection for 1 hour. Plasma was collected from the tail tip at 3 minutes and 1 hour after LiCl injection. Lithium concentration in urine and plasma samples was determined by inductively coupled plasma–mass spectrometry (Thermo iCAP RQ ICP-MS) at the Environmental and Complex Analysis Laboratory at the University of California, San Diego. Plasma creatinine concentration was determined by isotope dilution liquid chromatography-tandem mass spectrometry at the O’Brien Center for Acute Kidney Injury Research at the University of Alabama at Birmingham (Birmingham, Alabama, USA) and urine creatinine by a kinetic modification of Jaffe’s reaction (Thermo Fisher Scientific). Fractional urinary lithium excretion was determined as: *FE_Li_* = (urine/plasma Li)/(urine/plasma creatinine), where plasma *Li* was the arithmetic mean of plasma lithium concentration measured at 3 minutes and 1 hour after LiCl injection.

### Micro-CT analysis of kidneys.

Kidneys were removed from mice, fixed and ethanol-dehydrated, and then scanned with a high-resolution micro-CT scanner. In the initial phase of the study, scans were acquired with a μCT40 (Scanco Medical) at 55 KeV and 6 μm cubic resolutions, and renal calcifications assessed with a threshold of 220, as previously described (50). In the latter phase of the study, the scanner was switched to a Quantum GX2 microCT and imaged at 45 kV/88uA with a 0.5 mm Al filter. Three reference kidney samples were scanned on both scanners and used to normalize intensity values across experiments.

### Micro–Fourier transform infrared spectroscopy.

Kidney sections (5 μm) were mounted on low-E glass slides (Kevley Technologies) for attenuated total internal reflection (ATR) imaging analysis. A serial section stained with Yasue silver replacement was used as a control section. Before infrared analysis, the control was visually examined with an Olympus white light microscope (×20 objective) to determine the areas of interest. Sections for ATR–micro–Fourier transform infrared spectroscopy (AT-FTIR) imaging were not stained. ATR infrared images were collected with a PerkinElmer Spectrum Spotlight 400 infrared imaging microscope interfaced to a PerkinElmer FTIR spectrometer, as described previously (8). Each image (400 × 400 μm area) had a spatial resolution of 1.56 μm/pixel and contained 65,746 infrared spectra collected at a spectral resolution of 8 wavenumbers. Each spectrum in the image is the average of 4 individual scans. The images were further processed using Spectrum Image software (PerkinElmer).

### Bone density measurements.

Dual-energy X-ray absorptiometry (DEXA, Lunar PIXImus, GE Medical Systems) was used to measure bone mineral density in anesthetized mice at the indicated ages. The region of interest was adjusted to capture the density of the femur, lumbar vertebrae, or the whole body.

### qPCR.

Whole tissue RNA was extracted with TRI Reagent (Sigma-Aldrich). First-strand cDNA iScript Reverse Transcription Supermix for RT-qPCR (Bio-Rad) was used for first-strand cDNA synthesis. mRNA quantitation was performed by Taqman assay using the ABI Prism 7900 HT Sequence Detection System (Applied Biosystems). Expression levels were normalized to ezrin. Threshold cycle (Ct) values were corrected for batch variation in amplification efficiency by using inter-run calibrator samples.

### Diuretic challenge experiment.

The protocol was modified from that described by Pei et al. (51). Twelve-week-old mice were acclimatized for 5 days in metabolic cages, then administered single intraperitoneal injections of furosemide (25 mg/kg body weight), hydrochlorothiazide (25 mg/kg body weight), or vehicle (2.5% NaOH in 0.9% NaCl) in a total volume of 10 mL per gram body weight. Four-hour collections of urine were taken at baseline, and immediately after each injection of diuretic or vehicle. During the protocol, mice were fed a gel-based diet (DietGel Recovery, ClearH_2_O) to ensure adequate fluid intake.

### Bulk chemical analysis of dissected kidney regions.

Our method was adapted from protocols developed by Schmidt-Nielsen (52, 53) and Fenton (52) to determine sodium and urea concentrations in rodent kidney tissues. 16- to 17-week-old mice were first anesthetized and the kidney excised, decapsulated, and cut into a block to expose a midcoronal face. This was then sectioned perpendicular to the corticomedullary axis into pieces of tissue representing the papilla, the cortex, and the segment of tissue in between, which included the entire outer medulla, and the base of the inner medulla. These were blotted on Whatman filter paper, transferred to microcentrifuge tubes, and weighed. The time from excision of the whole kidney to isolation of all segments was approximately 2 minutes. Assuming, very conservatively, that tissue Ca^2+^ diffuses at the same rate as in free solution (diffusion coefficient at 4°C ~ 5 × 10^–6^ cm^2^/s; refs. 54, 55), the time required for 50% of Ca^2+^ to diffuse 1 mm is greater than 30 minutes (56). So there should be reasonable preservation of any Ca^2+^ gradient within the time frame of the dissection. The tissue samples were then air-dried at 60°C overnight over desiccant and reweighed.

To determine total calcium content by the “acid ash” method, dessicated tissue fragments were completely dissolved by adding 25 mL of 35% nitric acid and 35% perchloric acid at 85°C, which generally took about 1 hour (57), then diluted in 75 mL water for assay. To determine diffusible calcium content, dessicated tissue fragments were suspended in 25 mL deionized water, heated to 90°C for 3 minutes, and incubated at 4°C for 18 hours to allow diffusion of calcium out of the tissue, and the aqueous solution taken for assay. Calcium concentration was then assayed by a phenolsulfonphthalein-based colorimetric method (Quantichrom, BioAssay Systems).

### LA-ICP-TOFMS.

Kidneys harvested from 6-week-old mice were snap-frozen in OCT using liquid nitrogen, sectioned into 10 μm slices, and stored in −80°C. Elemental images were collected by LA-ICP-TOFMS using an imageBIO266 laser ablation laser ablation system interfaced with an Vitesse time-of-flight ICP-MS at the Biomedical National Elemental Imaging Resource. Spatial distributions of calcium, sodium, and phosphorus were determined from the ^44^Ca, ^23^Na, and ^31^P isotope maps, respectively. Concentration profiles along the corticomedullary axis were determined from line scans at 100 μm intervals. Complete details are provided in Supplemental Methods and Supplemental Figure 8.

### Statistics.

Statistical analyses were performed with R version 4.2.1. Data for continuous variables are presented as mean ± SE. Multi-way ANOVA was used to test for differences in multigroup experiments. Linear mixed-effects models (LMMs) with random intercept were used for experiments with repeated measurements because this method is robust to different numbers of measurements in different subjects and to missing values, and can generate interpretable coefficients. *P* values less than 0.05 were considered significant.

### Study approval.

All animal experiments were conducted in accordance with the NIH *Guide for the Care and Use of Laboratory Animals* (National Academies Press, 2011) following protocol review and approval by the University of Kansas Medical Center and the Veterans Administration San Diego Healthcare System Institutional Animal Care and Use Committee.

### Data availability.

Values for all data points in graphs are reported in the Supporting Data Values file.

## Author contributions

ASLY, CVB, and DN designed the research study. CVB, DN, OAG, SK, YCK, NL, HG, PSR, AJS, MNB, and TP conducted the experiments. ASLY, DN, VV, APE, TP, BPJ, and JCW analyzed the data. ASLY and CVB drafted the manuscript, and all the authors reviewed and edited the manuscript.

## Funding support

This work is the result of NIH funding, in whole or in part, and is subject to the NIH Public Access Policy. Through acceptance of this federal funding, the NIH has been given a right to make the work publicly available in PubMed Central.

- NIH, R01 DK115727, to ASLY.
- NIH, University of Alabama at Birmingham/University of California, San Diego O’Brien Center of Acute Kidney Injury Research, U54 DK137307.
- Department of Veterans Affairs, to VV.
- National Institute of General Medical Sciences (NIGMS), R24GM141194 and 1S10OD032352-01, to Dartmouth Biomedical National Elemental Imaging Resource.

## Supplementary Material

Supplemental data

Unedited blot and gel images

Supporting data values

## Version 1 10/09/2025

In-Press Preview

## Version 2 12/01/2025

Electronic publication
