# Supplementary material for: Kidney-specific claudin-2 deficiency leads to medullary nephrocalcinosis in mice
Source: J Clin Invest. 2025 Oct 9;135(23):e197807. doi: 10.1172/JCI197807 (PMC12646660; doi:10.1172/JCI197807)
Supplement: Supplemental data [file jci-135-197807-s011.pdf]

SUPPLEMENTAL MATERIALS
Behm *et al.*

CONTENTS

Supplemental Methods

Supplemental Tables
Suppl. Table 1. Relationship of severity of hypercalciuria to development of nephrocalcinosis in genetic mouse models with impaired renal tubule calcium reabsorption

Supplemental Table 2. Linear mixed model for log-transformed element concentration as a function of corticomedullary distance

Supplemental Table 3. Relationship of severity of hypercalciuria to development of nephrocalcinosis in genetic rodent models with impaired renal tubule calcium reabsorption

Supplemental Figures

Suppl. Fig. S1. Targeting strategy for generation of *Cldn2* floxed conditional knockout mice.

Suppl. Fig. S2. Assessment of Cre recombination efficiency.

Suppl. Figure S3. Bulk tissue analysis of calcium content in older mice.

Suppl. Figure S4. ^{23}Na spatial maps by LA-ICP-TOF-MS in coronal kidney sections.

Suppl. Figure S5. ^{44}Ca spatial maps by LA-ICP-TOF-MS in coronal kidney sections.

Suppl. Figure S6. ^{31}P spatial maps by LA-ICP-TOF-MS in coronal kidney sections.

Suppl. Figure S7. Comparison of LA-ICP-TOF-MS spatial maps for 10 elements in a control mouse kidney.

Suppl. Figure S8. Method for quantitation of corticomedullary elemental concentration profile.

Supplemental References

SUPPLEMENTAL METHODS

Elemental Imaging
Kidney sections were analyzed via laser ablation inductively coupled plasma time of flight mass spectrometry (LA-ICP-TOF-MS) using an imageBIO266 laser ablation (LA) instrument (Element Scientific Lasers, Omaha, NE) interfaced to a Nu Instruments Vitesse (Wrexham, UK) ICP-TOF-MS via a Direct Concentric Injector. Laser ablation used a solid state 266 nm laser, which couples with biological materials but not glass slides. The laser was operated at a fluence of 3.5 J cm-2 with a 200 Hz laser frequency. Low resolution images used a 6 μm circular spot, and high resolution images of the corticomedullary axis were collected with a 2 μm circular spot. The ICP-TOF-MS was tuned to operate with individual laser pulse widths of 5-6 milliseconds, using a nebulizer flow of 1050-1250 (ml/min) and helium flow through the chamber and imaging cup of 225-300 and 200-225 ml/min respectively. The ICP-TOF-MS uses a segmented reaction cell with H_2 flow of 4 ml/min and He at a flow of 15 ml/min. The mass range monitored was from sodium at mass 23 to selenium at mass 80. Instrumentation was tuned daily to optimize response sensitivity and laser single pulse rate.

Quantification
LA and ICP-TOF-MS data were unified using Iolite software (1) which also performs background correction (using the signal from a gas only blank between ablation for each line using a linear spline function) and converts raw counts into counts per second. Counts per second data were quantified into parts per million (ppm) using a custom made micro-spotted gel standard calibration series (BIO-logi-CAL, Teledyne, Europe) (2) consisting of six concentrations of elements of interest, ranging from 0 to 4000 femtograms total per spot. Data reduction was performed in the Iolite 4 software package using the 3D Trace elements scheme. Gel standards were ablated before and after every specimen. In addition, an in-house secondary check standard consisting of 10 μm thick cryo-sectioned homogenized bovine heart tissue was analyzed with every specimen.

Post-processing analysis
Elemental images were processed using “ScaleBarOn,” a custom Python-based pipeline developed for comparative scaling of multiple elemental image, spatial alignment, and automated composite elemental image generation. The code calculates 99th percentile values for comparative scaling of multiple specimens for each element, supports ppm and CPS inputs, and allows batch export of elemental images and 99th percentile summaries in standard visually uniform color schemes.

Corticomedullary line profiles of 100 μm width were collected from low resolution elemental images of Ca and Na, originating from the papillae to the cortex (Supplemental Fig. S3). For statistical analysis, linear mixed models were generated with log-transformed element concentration as the dependent variable, corticomedullary distance as a repeated measure, additional fixed effects for genotype and sex, and random effects for the intercept and slope. *P* values for the fixed effects were determined from t-statistics using Satterthwaite's method for denominator degrees of freedom, as implemented in the lmerTest R package.

High resolution image and colocalization studies
For colocalization studies, adjacent serial sections were stained with hematoxylin and eosin, or immunofluorescently labeled with mouse anti-aquaporin 1 (Abcam, monoclonal antibody ab9566) and rabbit polyclonal anti-aquaporin 2 (gift of Dr. Mark Knepper). The high resolution elemental images were then co-registered with the immunohistochemical images using a 3-point coregistration process in the Iolite software package.

Supplemental Table 1. Ratio of element concentration in papillary tip to concentration in superficial cortex

Element	Cre–		Cre+	
	Female	Male	Female	Male
^{23}Na	6.06 (1.49)	5.16 (1.36)	6.69 (2.61)	7.12 (5.74)
^{44}Ca	3.23 (0.29)	3.47 (0.63)	4.02 (1.10)	26.39 (44.98)

Supplemental Table 2. Linear mixed model for log-transformed element concentration as a function of corticomedullary distance

	^{23}Na			^{44}Ca		
	Estimate (SE)	exp(estimate)*	*P* value	Estimate (SE)	exp(estimate)*	*P* value
Intercept	4.449 (0.369)	85.52		3.433 (0.179)	30.96	
Distance (mm)	0.394 (0.052)	1.483	<0.0001	0.132 (0.068)	1.141	0.069
Genotype (Cre+)	-0.149 (0.404)	0.861	0.72	0.107 (0.196)	1.113	0.59
Sex (male)	0.692 (0.404)	1.997	0.11	-0.228 (0.196)	0.796	0.26
Distance*genotype	0.103 (0.057)	1.109	0.091	0.191 (0.074)	1.211	0.020
Distance*sex	-0.086 (0.057)	0.918	0.15	0.052 (0.074)	1.053	0.50

Reference group is Cre–, female; distance is measured from the cortical surface

SE, standard error

*Exponential of the model coefficients. For the intercept, this represents concentration in ppm. For fixed effects, this represents the proportionate change in concentration for a unit change in distance, or for the indicated categorical group relative to the reference group.

Supplemental Table 3. Relationship of severity of hypercalciuria to development of nephrocalcinosis in genetic rodent models with impaired renal tubule calcium reabsorption

Gene modification	Affected nephron segment	Hypercalciuria[a]	Nephrocalcinosis	Age[b]	References
Cldn2, global KO	PT	2-fold	Dense papillary deposits of hydroxyapatite	6 months	(3)
Cldn2, kidney-specific KO	PT	None	Papillary deposits of hydroxyapatite	8 (female) to 12 (male) months	Currently described mouse model
Cldn16, siRNA KD	TALH	3- to 4-fold	Minimal deposits surrounding BM of medullary tubules	6 months	(4)
Cldn16, global KO	TALH	3-fold (neonate) to 11-fold (juvenile)	None	11 months	(5)
Cldn19, siRNA KD	TALH	3-fold	ND	ND	(6)
Nkcc2, global KO	TALH	4-fold	Mild tubule calcification	5 months	(7)
Trpv5, global KO	DCT	6-fold	None	ND	(8, 9)
Trpv5, S682P/+	DCT	6-fold	None	ND	(9)
Trpv5, S682P/S682P	DCT	24-fold	None	ND	(9)
Genetic hypercalciuric stone-forming (GHS) rats	Unknown	8- to 10-fold	None	~6 months	(10)

[a]Fold increase in urine calcium excretion rate or fractional excretion of calcium, compared to controls
[b]Age at which mice examined for nephrocalcinosis (earliest age if found)
KO, knockout; KD, knockdown; PT, proximal tubule; TALH, thick ascending limb of Henle; DCT, distal convoluted tubule; BM, basement membrane; ND, not described

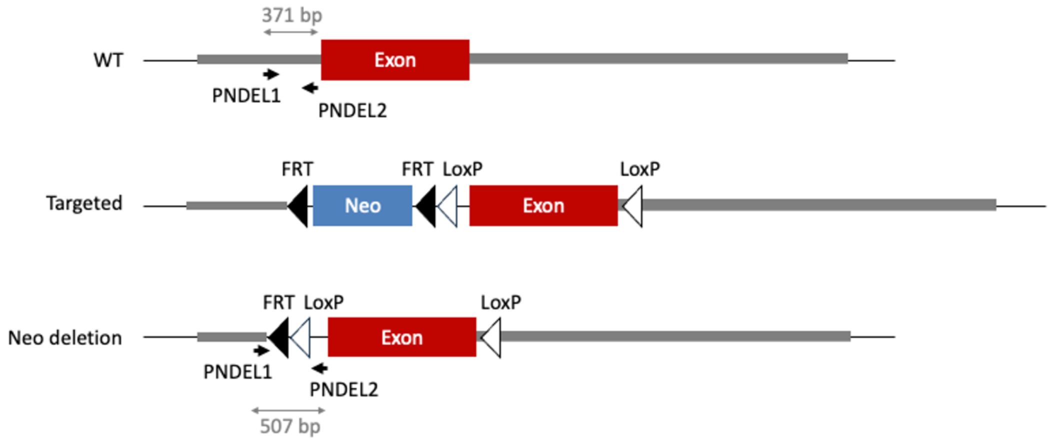

Supplemental Figure S1. Targeting strategy for generation of *Cldn2* floxed conditional knockout mice. A targeting vector with the neomycin resistance gene (Neo) was designed to target the single coding exon of mouse *Cldn2* (exon 2, ENSMUSE00000377520) in HF4 embryonic stem cells, which have a hybrid background (129/SvEv x C57Bl/6) and express Flp recombinase. Clones were selected with G418 antibiotic, and the Neo cassette, which was flanked by Flp recognition target (FRT) sequences, was deleted during clonal expansion, leaving behind a pair of LoxP sites flanking Cldn2. Genotyping primers, PNDEL1 (5'-CCTTCTCTTTCCGAGTATGGGC-3') and PNDEL2 (5'- GGCAGTCAGACAAAGGCAGG -3'), span a 371 bp region in the wild-type (WT) genome, but 507 bp in the correctly targeted and Neo-deleted mice.

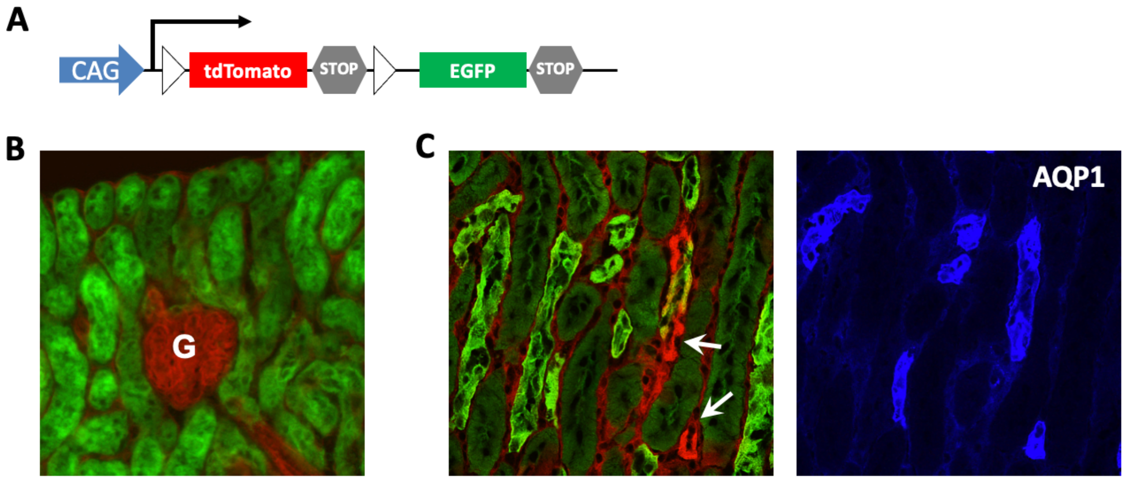

Supplemental Figure S2. Assessment of Cre recombination efficiency. **a.** Genetic scheme for mT/mG mice. Cell membrane-localized tdTomato (red fluorescence) is normally expressed constitutively under the control of a CMV enhancer/beta-actin promoter (CAG). A transcriptional stop signal prevents read-through downstream. In cells efficiently expressing Cre recombinase, tdTomato and the stop signal are excised, and replaced by expression of membrane-localized EGFP (green fluorescence). Fluorescence imaging of unfixed frozen sections of cortex (**b**) and outer medulla (**c**) from doxycycline-treated *Cldn2* floxed, Pax8-LC1 inducible knockout mice. In the cortex, efficient Cre recombination is observed in proximal tubules (green) and absent from glomeruli (red, G). In the outer medulla, thin descending limbs, identified by staining for AQP1 (blue) show incomplete recombination, as evidenced by patches of red fluorescence (arrows).

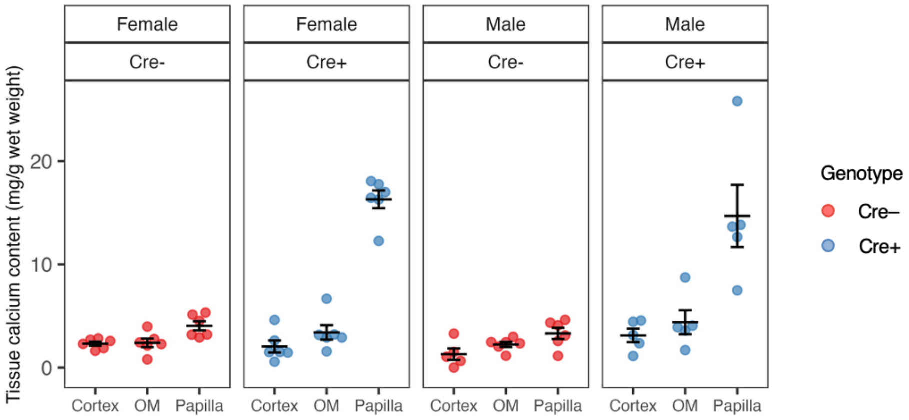

Supplemental Figure S3. Bulk tissue analysis of calcium content in older mice. Cortex, medulla (OM) and papilla were dissected from 26 week-old kidney-specific *Cldn2* knockout (Cre+) and control (Cre–) mice. Corresponding kidney segments from both kidneys (the entire 2 papillae or approximately 30 mg of each cortex or medulla) were transferred into microcentrifuge tubes, the weight determined, and then snap-frozen in liquid nitrogen and stored at −80°C for further analysis. Cold PBS was added (3.33 µL per mg of tissue) and the thawed tissue homogenized using a disposable pestle followed by sonication on ice. Homogenates were centrifuged at 16,000 x g for 10 minutes at 4°C and supernatants carefully collected. Calcium levels were measured in supernatants using the QuantiChrom Calcium Assay Kit (BioAssay Systems, Hayward, CA, USA). $P < 0.001$ for main effects of genotype and segment and the genotype x segment interaction by three-way ANOVA.

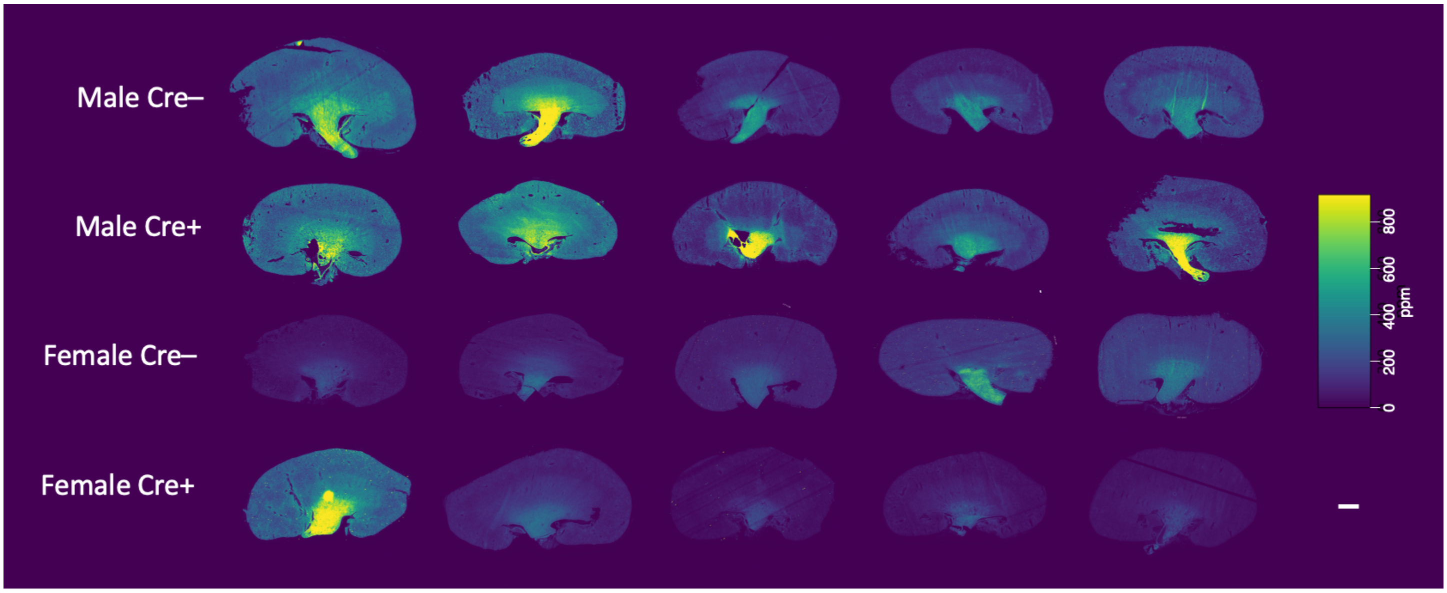

Supplemental Figure S4. ^{23}Na spatial maps acquired at 6 µm resolution by LA-ICP-TOF-MS in coronal kidney sections of kidney-specific *Cldn2* knockout (Cre+) and control (Cre–) mice. The full dataset is shown at the same concentration scale. Concentration scale bar on the right, in units of parts per million (ppm) . The viridis color scale is used to ensure perceptual uniformity between color and grayscale, and visibility with color blindness. Dimension scale on lower right is shown for 1 mm.

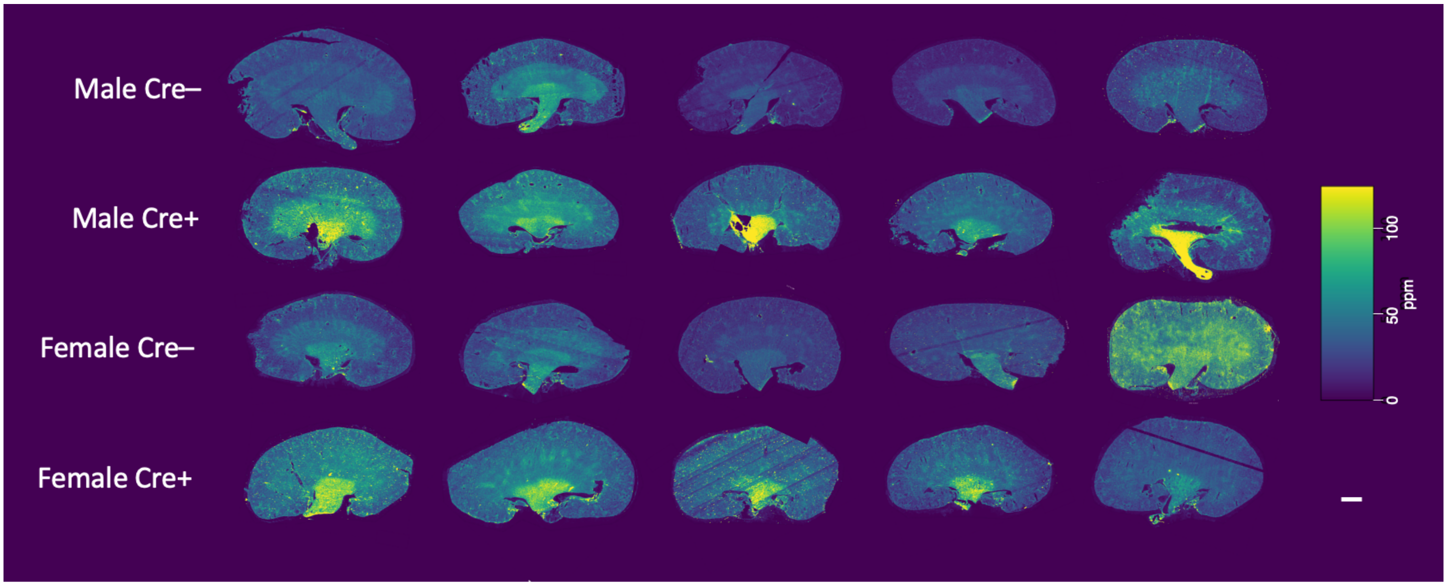

Supplemental Figure S5. ^{44}Ca spatial maps acquired at 6 µm resolution by LA-ICP-TOF-MS in coronal kidney sections of kidney-specific *Cldn2* knockout (Cre+) and control (Cre–) mice. The full dataset is shown at the same concentration scale. Concentration scale bar on the right, in units of parts per million (ppm) . The viridis color scale is used to ensure perceptual uniformity between color and grayscale, and visibility with color blindness. Dimension scale on lower right is shown for 1 mm.

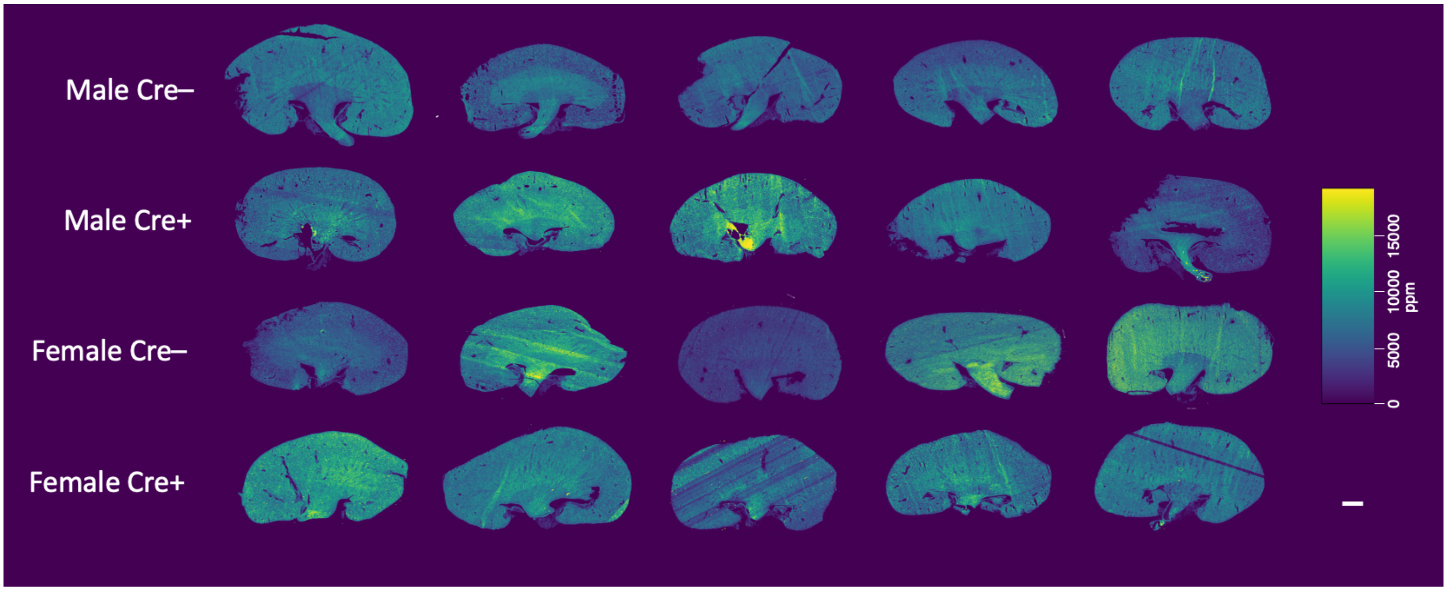

Supplemental Figure S6. ^{31}P spatial maps acquired at 6 µm resolution by LA-ICP-TOF-MS in coronal kidney sections of kidney-specific *Cldn2* knockout (Cre+) and control (Cre–) mice. The full dataset is shown at the same concentration scale. Concentration scale bar on the right, in units of parts per million (ppm) . The viridis color scale is used to ensure perceptual uniformity between color and grayscale, and visibility with color blindness. Dimension scale on lower right is shown for 1 mm.

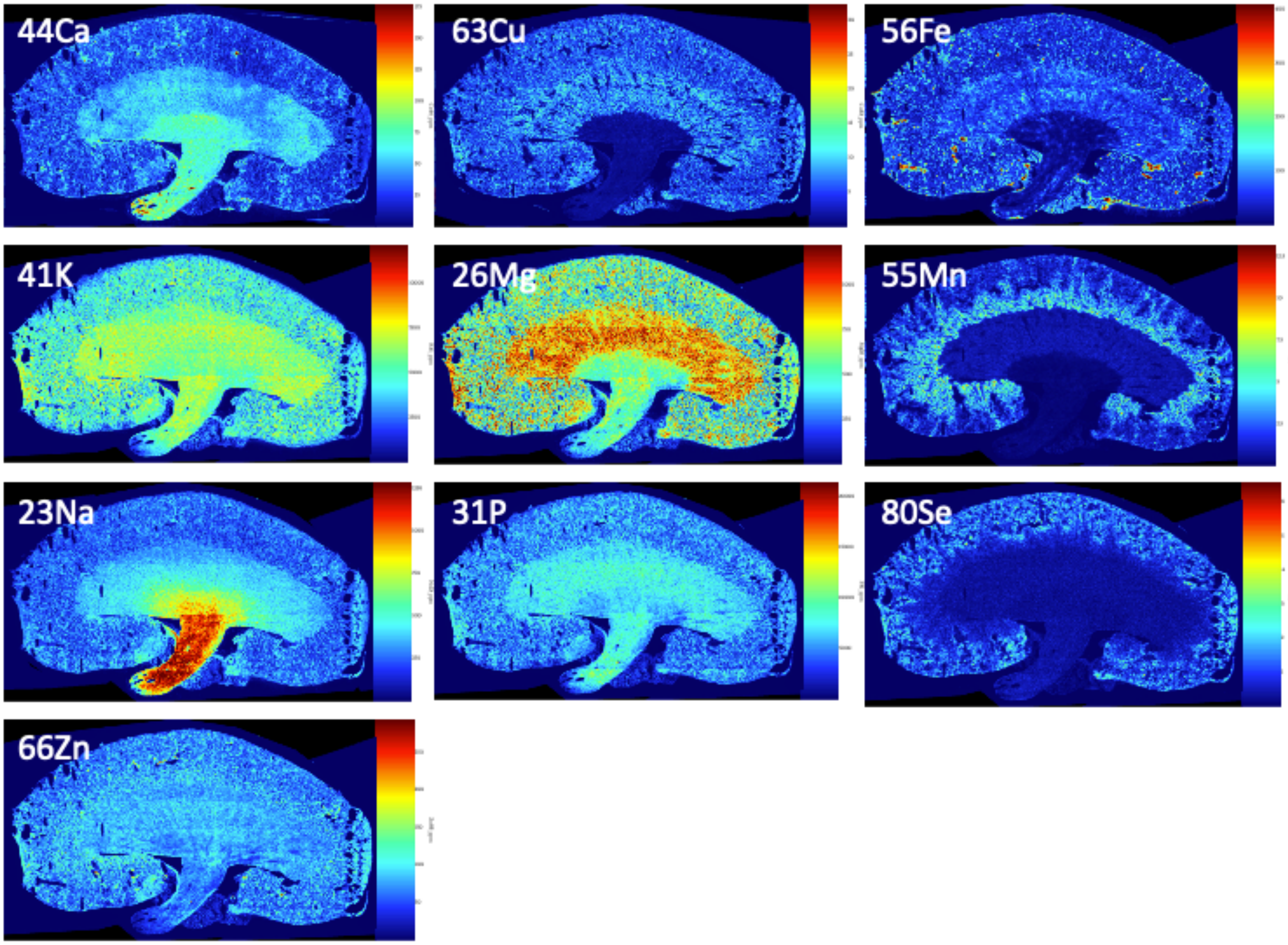

Supplemental Figure S7. Comparison of LA-ICP-TOF-MS spatial maps for 10 elements in a control mouse kidney. Color scale bar on the right of each panel depicts concentration in ppm. Even at low resolution, it is possible to discern not only differences in regional distribution between elements, but localization of some elements to tubule or interstitial compartments.

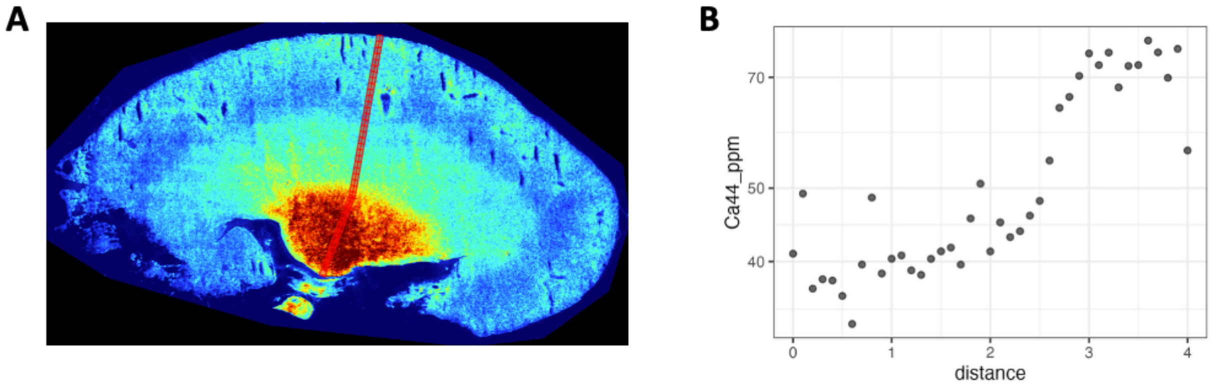

Supplemental Figure S8. Method for quantitation of corticomedullary elemental concentration profile. **A.** A straight or segmented line was drawn to delineate the corticomedullary axis and bisect the papillary tip (red). **B.** Average ^{44}Ca or ^{23}Na concentration was quantitated within serial 100 µm x 100 µm squares along the lines, log-transformed and plotted as a function of distance.

SUPPLEMENTAL REFERENCES

1. Paton C, Hellstrom J, Paul B, Woodhead J, and Hergt J. Iolite: Freeware for the visualisation and processing of mass spectrometric data. *Journal of Analytical Atomic Spectrometry.* 2011;26(12):2508-18.
2. Theiner S, Egger A, Keppler B, Heffeter P, Kornauth C, Theiner S, et al. Bioimaging and quantification of metal-based anticancer drugs using LA-ICP-MS. *Journal of Biological Inorganic Chemistry.* 2014;19:S681-S.
3. Curry JN, Saurette M, Askari M, Pei L, Filla MB, Beggs MR, et al. Claudin-2 deficiency associates with hypercalciuria in mice and human kidney stone disease. *J Clin Invest.* 2020;130(4):1948-60.
4. Hou J, Shan Q, Wang T, Gomes AS, Yan Q, Paul DL, et al. Transgenic RNAi depletion of claudin-16 and the renal handling of magnesium. *J Biol Chem.* 2007;282(23):17114-22.
5. Will C, Breiderhoff T, Thumfart J, Stuiver M, Kopplin K, Sommer K, et al. Targeted deletion of murine Cldn16 identifies extra- and intrarenal compensatory mechanisms of Ca2+ and Mg2+ wasting. *Am J Physiol Renal Physiol.* 2010;298(5):F1152-61.
6. Hou J, Renigunta A, Gomes AS, Hou M, Paul DL, Waldegger S, et al. Claudin-16 and claudin-19 interaction is required for their assembly into tight junctions and for renal reabsorption of magnesium. *Proc Natl Acad Sci U S A.* 2009;106(36):15350-5.
7. Takahashi N, Chernavvsky DR, Gomez RA, Igarashi P, Gitelman HJ, and Smithies O. Uncompensated polyuria in a mouse model of Bartter's syndrome. *Proc Natl Acad Sci U S A.* 2000;97(10):5434-9.
8. Hoenderop JG, van Leeuwen JP, van der Eerden BC, Kersten FF, van der Kemp AW, Merillat AM, et al. Renal Ca2+ wasting, hyperabsorption, and reduced bone thickness in mice lacking TRPV5. *J Clin Invest.* 2003;112(12):1906-14.
9. Loh NY, Bentley L, Dimke H, Verkaart S, Tammaro P, Gorvin CM, et al. Autosomal dominant hypercalciuria in a mouse model due to a mutation of the epithelial calcium channel, TRPV5. *PLoS One.* 2013;8(1):e55412.
10. Evan AP, Bledsoe SB, Smith SB, and Bushinsky DA. Calcium oxalate crystal localization and osteopontin immunostaining in genetic hypercalciuric stone-forming rats. *Kidney Int.* 2004;65(1):154-61.
