# Supplementary figures and images for: Kidney-specific claudin-2 deficiency leads to medullary nephrocalcinosis in mice
Source: J Clin Invest. 2025 Oct 9;135(23):e197807. doi: 10.1172/JCI197807 (PMC12646660; doi:10.1172/JCI197807)

Figure 1A

Figure 3A

Kidney

Colon

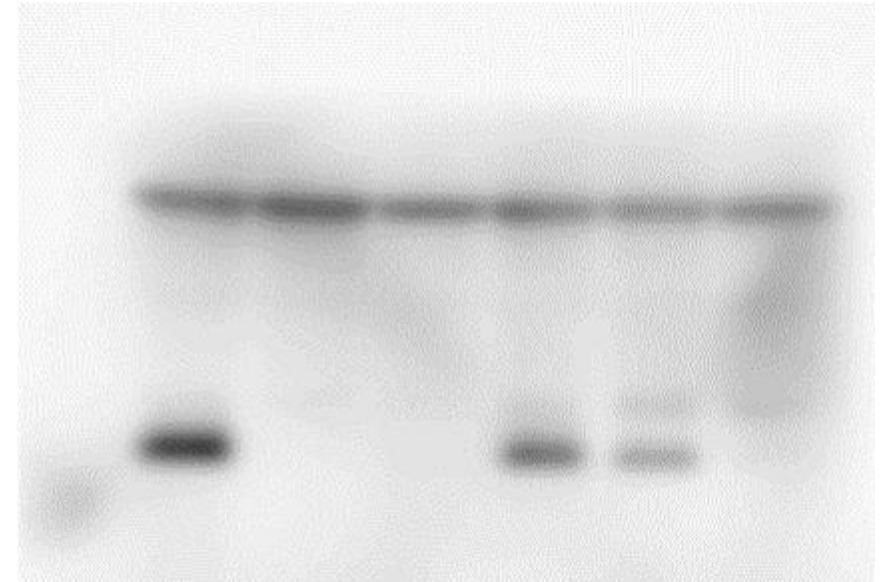

Mouse anti-claudin-2 (ThermoFisher Scientific #32-5600)

Supplement: Unedited blot and gel images [file jci-135-197807-s012.pdf]
